# Mortality rates and risk factors in 1412 Japanese patients with decompensated hepatitis C virus-related cirrhosis: a retrospective long-term cohort study

**DOI:** 10.1186/s12876-021-01770-0

**Published:** 2021-04-23

**Authors:** Shunichiro Fujiyama, Norio Akuta, Hitomi Sezaki, Mariko Kobayashi, Yusuke Kawamura, Tetsuya Hosaka, Masahiro Kobayashi, Satoshi Saitoh, Fumitaka Suzuki, Yoshiyuki Suzuki, Yasuji Arase, Kenji Ikeda, Hiromitsu Kumada

**Affiliations:** 1 Department of Hepatology, Toranomon Hospital, Okinaka Memorial Institute for Medical Research, 2-2-2 Toranomon, Minato-ku, Tokyo, 105-0001 Japan; 2 Liver Research Laboratory, Toranomon Hospital, Tokyo, Japan

**Keywords:** Decompensated HCV-related cirrhosis, Hepatocellular carcinoma, Long-term, Mortality, Natural history

## Abstract

**Background:**

Hepatitis C virus is the leading cause of liver cirrhosis and hepatocellular carcinoma in Japan. We aimed to examine the long-term (> 20 years) mortality and hepatocellular carcinoma rates and associated risk factors in 1412 Japanese patients with decompensated hepatitis C virus-related cirrhosis (Child–Pugh B or C).

**Methods:**

Cumulative survival and hepatocellular carcinoma rates were determined using Kaplan–Meier analysis. Independent risk factors were identified by multivariate analysis. A two-tailed *P-*value of < 0.05 was considered significant.

**Results:**

The patients were followed up for a median of 2 years (range 0.5–24.2 years). In total, 62.3%, 41.7%, 4.7%, and 68.3% of the patients had a history of hepatocellular carcinoma, ascites, hepatic encephalopathy, and esophageal varices, respectively. The 1-, 5-, 10-, and 20-year cumulative overall survival rates in the total cohort was 74.9%, 29.0%, 9.1%, and 1.4%, respectively. The 1-, 3-, 5-, and 10-year cumulative survival rates for patients without hepatocellular carcinoma were 93.1%, 54.4%, 18.2%, and 4.0%, respectively, and the corresponding cumulative post-decompensation hepatocellular carcinoma rates were 14.0%, 31.6%, 46.1%, and 66.2%, respectively. The independent risk factors for mortality were older age, Child–Pugh C cirrhosis, the presence of hepatocellular carcinoma, low estimated glomerular filtration rate, low serum sodium level, low platelet count, and high γ-glutamyl transferase and α-fetoprotein levels for all patients and older age, Child–Pugh C cirrhosis, and low estimated glomerular filtration rate for patients without hepatocellular carcinoma. Overall, 1035 patients (73.3%) died; the causes of death were liver failure with/without hepatocellular carcinoma, pneumonia, sepsis, cardiovascular disease, and non-hepatocellular carcinoma malignancies. The corresponding morality rates per person-year were 133.4, 59.9, 10.9, 10.6, 9.0, and 5.2, respectively.

**Conclusions:**

Among Japanese patients with decompensated hepatitis C virus-related cirrhosis, hepatocellular carcinoma is associated with poor prognosis. Our results highlight the importance of managing liver-related events, including hepatocellular carcinoma, in these patients.

**Supplementary Information:**

The online version contains supplementary material available at 10.1186/s12876-021-01770-0.

## Background

Liver cirrhosis is associated with high morbidity and mortality, accounting for approximately 1 million deaths annually worldwide [1]. In Japan, the hepatitis C virus (HCV) is the main cause of liver cirrhosis and hepatocellular carcinoma (HCC). Decompensated cirrhosis is a terminal liver disease whose complications include gastrointestinal variceal bleeding, infection, ascites, and hepatic encephalopathy [2–4]. Although liver transplantation markedly improves the survival of patients with decompensated cirrhosis, it is not widely used because of limited liver resources, high costs, and immunological transplantation responses [5]. In Japan, livers are mainly obtained from living donors, and only a small number obtained from brain-dead donors.

Hepatic dysfunction severely impairs a person’s ability to work and quality of life, and decompensated cirrhosis is still associated with a high economic burden. Furthermore, survival and mortality rates differ significantly among patients with decompensated cirrhosis [6]. In the study by Planas et al*.* [7], the probabilities of survival after diagnosis of liver cirrhosis without HCC, including compensated cirrhosis, were 81.8% at 1 year and 50.8% at 5 years. In patients with Child–Pugh B and C cirrhosis, they were 46.3% and 36.4% at 5 years, respectively. A systematic review based on 118 studies from 1983 to 2005 [8] showed that the median survival time in patients with decompensated cirrhosis, including those with etiologies aside from HCV, was < 2 years. The 2-year survival probability in patients with Child–Pugh C cirrhosis was 38%.

Medical decisions regarding the treatment of decompensated HCV-related cirrhosis require knowledge of its clinical course and prognosis. However, such knowledge is lacking, and there are no large-scale data analyses for Asians. Thus, the present study aimed to define the long-term natural course (> 20 years) of HCV-related cirrhosis after hepatic decompensation and to identify factors associated with HCC occurrence and mortality in Japan.

## Methods

### Study design and patients

We enrolled 1454 consecutive patients with decompensated HCV-related cirrhosis who were admitted to Toranomon Hospital between 1979 and 2020. We excluded patients with (1) underlying liver disease (e.g., other viral hepatitis or drug-induced liver disease), (2) systemic autoimmune diseases (e.g., systemic lupus erythematosus or rheumatoid arthritis), (3) metabolic diseases (e.g., hemochromatosis, α-1-antitrypsin deficiency, or Wilson disease), (4) a history of excessive alcohol intake, and (5) a history of interferon or direct-acting antiviral (DAA) treatment. Patients who received DAA treatment were excluded because this treatment was only recently approved (February 2019) for decompensated HCV-related cirrhosis in Japan.

The assessed decompensations of liver cirrhosis included ascites, jaundice, variceal bleeding, portal hypertensive gastrointestinal bleeding (PHGB), hepatic encephalopathy, and severe bacterial infection.

### Diagnosis and follow-up

The diagnosis of cirrhosis was based on liver biopsy findings and/or an unequivocal clinical and biochemical profile, a highly suggestive endoscopic examination, or typical imaging findings in agreement with current guidelines. Prior to the introduction of a diagnostic assay in 1989, HCV was diagnosed using previously stored serum. The date of the hepatic decompensation was defined as day 1, and all patients were followed until 6 months as the end of the observation period (April 2020) or until death, whichever came first. Follow-up comprised clinical assessment and standard liver biochemical tests biannually or more frequently as needed. Hepatic protective agents other than antiviral therapy and treatments for complications associated with liver cirrhosis (e.g., albumin or diuretics) were provided at the discretion of the attending physician.

Data on the following factors were collected from each patient: hepatic decompensations, HCC development, and death. Ascites was always confirmed using ultrasonography or paracentesis. Spontaneous bacterial peritonitis was diagnosed based on an ascitic fluid polymorphonuclear count of > 250 cell/mm^3^ and the absence of data suggesting secondary peritonitis [9]. The prognosis of patients with decompensated cirrhosis was evaluated using the Model for End-Stage Liver Disease (MELD) score, which incorporates serum bilirubin, creatinine, and the prothrombin time-international normalized ratio (PT-INR). The MELD score was calculated using the following formula: MELD score = (9.57 × loge creatinine [mg/dl]) + (3.78 × loge bilirubin [mg/dl]) + (11.20 × loge PT-INR) + 6.43 and then rounded to the nearest integer.

HCC was evaluated at admission via ultrasonography and measurement of α-fetoprotein (AFP) levels. Diagnosis was confirmed via histology and/or by two coincidental imaging techniques associated with elevated AFP levels [10]. Hepatic encephalopathy was defined as an episode of mental confusion clearly reported by the patient or a family member or disorientation detected by a physician. Confusion or coma before death from hepatic failure was excluded [11]. PHGB was diagnosed in accordance with the Baveno consensus criteria [12]. Death was considered to be caused by liver failure if progressive liver function deterioration was observed regardless of its severity or if death occurred within 6 weeks of PHGB [12]. Patients who died of conditions unrelated to liver disease and those who were lost to follow-up were censored at the time of death or at the time of dropout, respectively.

### Statistical analysis

Parameters that strongly correlated with other parameters were considered confounding factors and excluded from statistical analysis. The incidence of each event was analyzed during the period from the time of diagnosis of decompensated HCV-related cirrhosis to the last visit. The cumulative probability of HCC development and survival during follow-up was calculated using the Kaplan–Meier method, and the survival curves were compared using the log-rank test. Stepwise Cox regression analysis based on the backward selection method was used to identify independent predictive factors for mortality. Hazard ratios and 95% confidence intervals were also calculated. Variables that were statistically significant on univariate analysis were entered into the multivariate analysis to identify significant independent factors after conversion to categorical data of two simple ordinal numbers. The median value of each factor was set as the cutoff point. All statistical tests were performed using the Statistical Package for the Social Sciences (SPSS) software version 20.0 (SPSS Inc., Chicago, IL, USA). A two-tailed *P*-value of < 0.05 was considered significant.

## Results

### Clinical characteristics

**Fig. 1:**
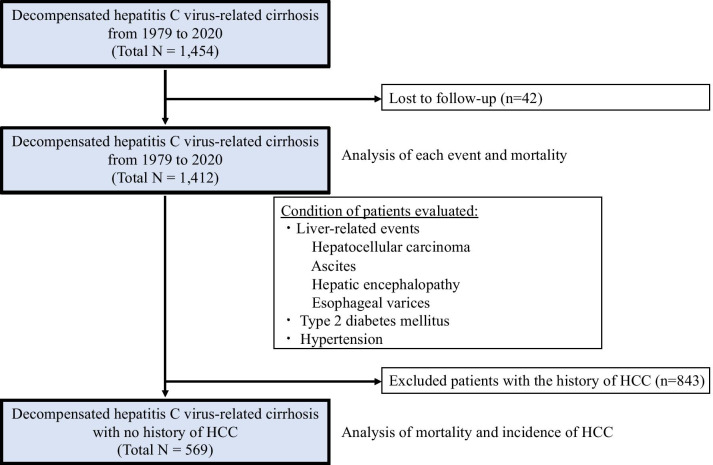
Patient selection flowchart. Of the 1454 patients with decompensated cirrhosis, 1412 patients, excluding 42 patients who were lost to follow-up, were evaluated. Furthermore, the cumulative survival rate and new liver carcinogenesis rate were examined in 569 patients, excluding the 843 patients with a history of HCC. HCC, hepatocellular carcinoma

The median follow-up was 2 years (range 0.5–24.2 years). Among the 1454 patients enrolled in our study, 42 (3.0%) were lost to follow-up owing to transfer to another hospital after their initial hepatic decompensation (Fig. 1). The baseline clinical and laboratory features of the final 1412 patients are shown in Table 1. There were 776 men and 636 women, and the median age was 68 years, with 64% of the patients aged > 65 years. At the time of clinical diagnosis of decompensated HCV-related cirrhosis, 843 (59.7%), 589 (41.7%), 66 (4.7%), and 965 (68.3%) patients had a history of HCC, ascites, hepatic encephalopathy, and esophageal varices, respectively. Liver transplant cases were not included in this study.

**Table 1:** Patient characteristics at the time of diagnosis of decompensated HCV-related cirrhosis (n = 1412 patients)

Factor	Value
*Clinicodemographic*	
Sex (male/female)	776/636
Age, years^a^	68 (31–97)
Child–Pugh classification (B/C)	1313/99
History of HCC (no/yes)	569/843
BCLC stage^b^, n (A/B/C/D)	235/85/16/12
Ascites (no/yes)	823/589
Hepatic encephalopathy (no/yes)	1346/66
Esophageal varices (no/yes)	447/965
Type 2 diabetes mellitus (no/yes)	1051/361
Hypertension (no/yes)	1022/390
*Laboratory parameters*^a^	
Serum albumin, g/dL [N = 1412]	3.0 (1.4–4.2)
Serum aspartate aminotransferase, U/L [N = 1412]	64 (5–5942)
Serum alanine aminotransferase, U/L [N = 1412]	42 (1–1306)
γ-glutamyl transpeptidase, U/L [N = 1407]	50 (2–8184)
Serum total bilirubin, mg/dL [N = 1412]	1.4 (0.2–31.1)
Serum creatinine, mg/dL [N = 1412]	0.8 (0.3–17.0)
eGFR, mL/min [N = 1412]	77.8 (2.4–220.5)
Sodium, mEq/L [N = 1388]	140 (113–153)
Ammonia, μg/dL [N = 678]	58 (4–415)
Platelet count, × 10^3^/mm^3^ [N = 1412]	8.2 (0.7–61.0)
Prothrombin time, % [N = 1412]	69.1 (7.6–117.4)
α-fetoprotein, μg/L [N = 1373]	34.0 (0.8–13,597,000)
MELD score^c^, n [N = 969]	7 (-5–35)

eGFR, estimated glomerular filtration rate; HCC, hepatocellular carcinoma; HCV, hepatitis C virus

^a^Median (range). All other data are presented as the number of patients

^b^The BCLC stage (A/B/C/D) could be examined in 348 patients

^c^The MELD score could be examined in 969 patients

As treatment for complications of decompensated cirrhosis, diuretics for ascites were given to 625 patients (44.3%); branched chain amino acid preparations as nutritional therapy were given to 267 patients (18.9%) with a serum albumin level of ≤ 3.5 g/dL; non-absorbable synthetic disaccharides and rifaximin (an intestinal non-absorbable antibacterial drug) for hepatic encephalopathy were given to 141 patients (10.0%); and endoscopic variceal ligation to prevent variceal bleeding was performed in 407 (28.8%).

### Mortality and HCC rates

**Fig. 2:**
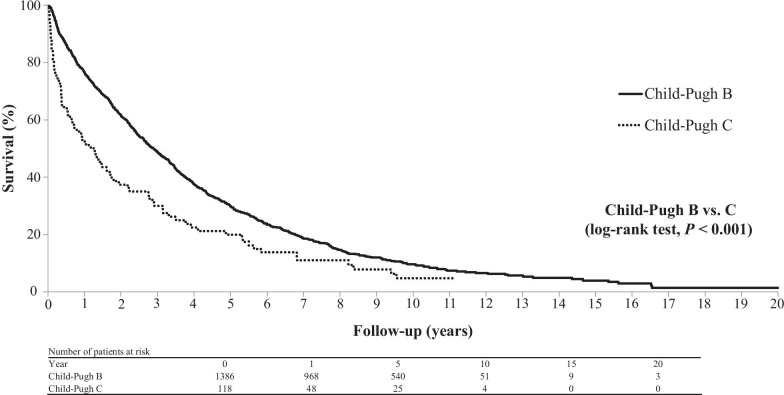
Cumulative survival rates over a 20-year period in the 1412 patients with decompensated HCV-related cirrhosis. The rates differed significantly between patients with Child–Pugh B and C cirrhosis (log-rank test, *P* < 0.001). HCV, hepatitis C virus

**Fig. 3:**
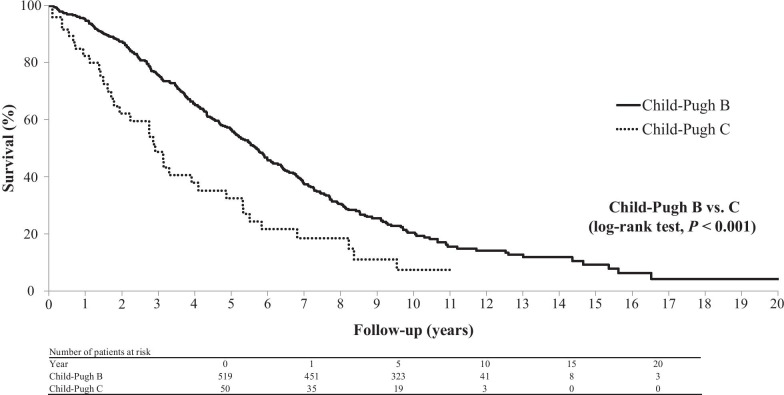
Cumulative survival rates over a 20-year period in patients with decompensated HCV-related cirrhosis without HCC. The rates differed significantly different between patients with Child–Pugh B and C cirrhosis (log-rank, *P* < 0.001). HCC, hepatocellular carcinoma; HCV, hepatitis C virus

In the total population, the 1-, 3-, 5-, 10-, 15-, and 20-year cumulative survival rates after the diagnosis of decompensated HCV-related cirrhosis were 76.7%, 48.7%, 30.7%, 10.5%, 4.5%, and 1.6% for patients with Child–Pugh B cirrhosis and 51.4%, 27.3%, 19.2%, 6.7%, 0.0%, and 0.0% for patients with Child–Pugh C cirrhosis, respectively (Fig. 2). In the 569 patients without HCC, they were 95.0%, 75.6%, 56.7%, 20.4%, 9.2%, and 4.2% in the Child–Pugh B group and 82.4%, 48.7%, 32.5%, 7.4%, 0.0%, and 0.0% in the Child–Pugh C group, respectively (Fig. 3). There was a significant difference in survival rates between the Child–Pugh B and C groups in both the entire cohort and in the cohort of patients without HCC (log-rank test, *P* < 0.001 in both instances).

**Fig. 4:**
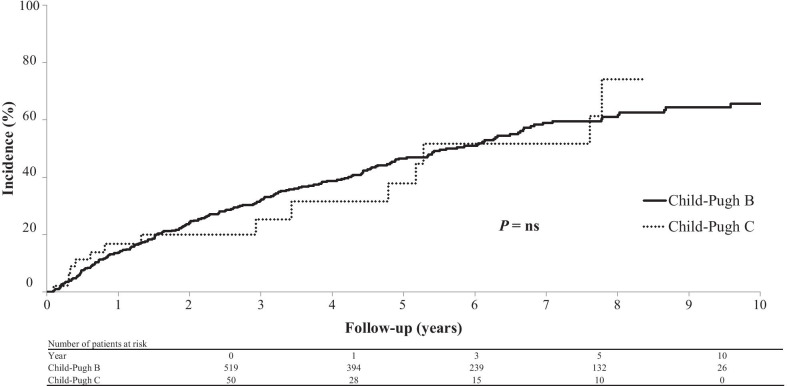
Cumulative incidence of HCC in patients without HCC at the time of diagnosis of cirrhosis. The rates were not significantly different between patients with Child–Pugh B and C cirrhosis. HCC, hepatocellular carcinoma; ns, not significant

Among the 569 patients without HCC at the time of diagnosis of decompensated cirrhosis, 233 patients (40.9%) developed new HCC during the follow-up. The cumulative HCC rate was 13.8% at 1 year, 32.0% at 3 years, 46.6% at 5 years, and 65.6% at 10 years in the Child–Pugh B group and 16.8% at 1 year, 25.3% at 3 years, and 37.8% at 5 years in the Child–Pugh C group. The difference in the cumulative HCC rate between the groups was not significant (Fig. 4).

### Factors associated with mortality

The clinicopathological features included in the survival analysis are listed in Table 1. The parameters (e.g., ascites, hepatic encephalopathy, albumin, total bilirubin, creatinine, ammonia, and prothrombin time) that strongly correlated with other parameters were considered confounding factors and excluded from statistical analysis. Of the remaining parameters, ten were significantly correlated with mortality in the entire cohort on univariate analysis: sex, age, Child–Pugh classification, a history of HCC, aspartate aminotransferase level, γ-glutamyl transpeptidase (GGT) level, estimated glomerular filtration rate (eGFR), serum sodium level, platelet count, and AFP level (Table 2). Multivariate analysis identified eight risk factors that significantly and independently influenced mortality: age ≥ 68 years (*P* < 0.001), Child–Pugh C cirrhosis (*P* < 0.001), a history of HCC (*P* < 0.001), GGT level ≥ 50 U/L (*P* < 0.001), eGFR < 77.8 mL/min (*P* < 0.001), serum sodium level < 140 mEq/L (*P* = 0.0022), platelet count < 8.2 × 10^3^/mm^3^ (*P* = 0.0024), and AFP level ≥ 33.9 μg/L (*P* < 0.001).

**Table 2:** Predictive factors of mortality in the 1412 patients with decompensated HCV-related cirrhosis

Factor	Category	Univariate analysis			Multivariate analysis		
		HR	95% CI	*P*-value^a^	HR	(95% CI)	*P*-value^a^
*Demographic*							
Sex	Male	1			1		
	Female	1.34	1.19–1.5	< 0.001	0.90	0.78–1.03	0.1174
Age, years	< 68	1			1		
	≥ 68	1.78	1.58–2.01	< 0.001	1.70	1.48–1.95	< 0.001
Child–Pugh classification	B	1			1		
	C	1.66	1.34–2.07	< 0.001	1.98	1.54–2.54	< 0.001
History of HCC	No	1			1		
	Yes	3.06	2.68–3.49	< 0.001	2.61	2.27–3.01	< 0.001
Esophageal varices	No	1					
	Yes	1.08	0.93–1.29	0.4592			
Type 2 diabetes mellitus	No	1					
	Yes	1.10	0.96–1.26	0.1792			
Hypertension	No	1					
	Yes	1.13	1.00–1.29	0.0581			
*Laboratory*							
Serum AST, IU/L	< 2 × ULN	1			1		
	≥ 2 × ULN/L	1.19	1.06–1.34	0.0034	0.88	0.77–1.00	0.0502
Serum ALT, IU/L	< 2 × ULN	1					
	≥ 2 × ULN	1.13	0.95–1.35	0.1792			
γ-GGT, U/L	< 50	1			1		
	≥ 50	1.15	1.02–1.29	0.0214	1.37	1.20–1.56	< 0.001
eGFR, mL/min	≥ 77.8	1			1		
	< 77.8	1.57	1.39–1.77	< 0.001	1.29	1.13–1.47	< 0.001
Sodium, mEq/L	≥ 140	1			1		
	< 140	1.45	1.29–1.64	< 0.001	1.29	1.13–1.47	0.0022
Platelets, × 10^3^/mm^3^	≥ 8.2	1			1		
	< 8.2	1.36	1.21–1.53	< 0.001	1.21	1.07–1.38	0.0024
AFP, μg/L	< 33.9	1			1		
	≥ 33.9	1.43	1.27–1.61	< 0.001	1.28	1.13–1.46	< 0.001

AFP, alpha-fetoprotein; ALT, alanine aminotransferase; AST, aspartate aminotransferase; CI, confidence interval; eGFR, estimated glomerular filtration rate; γ-GGT, γ-glutamyl transpeptidase; HCC, hepatocellular carcinoma; HCV, hepatitis C virus; HR, hazard ratio

^a^Significance was determined using the Cox proportional hazard model. The ten variables that were significant in the univariate analysis were entered into the multivariate analysis to identify significant independent factors. Multivariate analysis was performed using the significant factors in univariate analysis (P < 0.05)

In the 569 patients without HCC, four parameters significantly correlated with mortality on univariate analysis: age, Child–Pugh classification, eGFR, and AFP level (Table 3). Multivariate analysis identified three independent risk factors for mortality: age ≥ 65 years (*P* < 0.001), Child–Pugh C cirrhosis (*P* < 0.001), and eGFR < 84.0 mL/minute (*P* < 0.001).

**Table 3:** Predictive factors of mortality in the 569 patients with decompensated HCV-related cirrhosis without HCC

Factor	Category	Univariate analysis			Multivariate analysis		
		HR	95% CI	*P*-value^a^	HR	(95% CI)	*P*-value^a^
*Demographic*							
Sex	Male	1					
	Female	1.18	0.96–1.46	0.1176			
Age, years	< 65	1			1		
	≥ 65	1.71	1.38–2.12	< 0.001	1.81	1.44–2.26	< 0.001
Child–Pugh classification	B	1			1		
	C	1.92	1.35–2.72	< 0.001	1.98	1.35–2.90	< 0.001
Esophageal varices	No	1					
	Yes	1.12	0.95–1.37	0.3230			
*Laboratory*							
eGFR, mL/min	≥ 84	1			1		
	< 84	1.68	1.36–2.08	< 0.001	1.69	1.36–2.11	< 0.001
Sodium, mEq/L	≥ 141	1					
	< 141	1.20	0.97–1.48	0.0999			
AFP, μg/L	< 18.5	1					
	≥ 18.5	0.76	0.61–0.94	0.123			

AFP, alpha-fetoprotein; CI, confidence interval; eGFR, estimated glomerular filtration rate; HCC, hepatocellular carcinoma; HCV, hepatitis C virus; HR, hazard ratio

^a^Significance was determined using the Cox proportional hazard model. The four variables that were significant in the univariate analysis were entered into the multivariate analysis to identify significant independent factors

### Clinical characteristics of patients who died during the study period

Overall, 1035 patients (73.3%) died during the study period. Their clinical features are shown in Table 4. Among these patients, 564 (39.9%), 253 (17.9%), 46 (3.3%), 45 (3.2%), 38 (2.7%), and 22 (1.6%) died of liver failure with HCC, liver failure without HCC, pneumonia, sepsis, cardiovascular disease, and malignancy events excluding HCC, respectively. The corresponding mortality rates per 1000 person-years were 133.4, 59.9, 10.9, 10.6, 9.0, and 5.2, respectively.

**Table 4:** Cause of death in the 1412 patients with decompensated HCV-related cirrhosis

Cause of death	n/N (%)^a^	1000 person-years
Overall	1035/1412 (73.3)	244.9
Liver failure with HCC	564/1412 (39.9)	133.4
Liver failure without HCC	253/1412 (17.9)	59.9
Pneumonia	46/1412 (3.3)	10.9
Sepsis	45/1412 (3.2)	10.6
Cardiovascular disease	38/1412 (2.7)	9.0
Malignancy events other than HCC	22/1412 (1.6)	5.2
Unknown	67/1412 (4.7)	15.9

HCC, hepatocellular carcinoma; HCV, hepatitis C virus

^a^n, number of events; N, number of patients

### Prognosis according to the MELD score

The MELD score could be examined in 969 patients. Those with a MELD score of ≤ 7 points had a better prognosis than did those with higher scores Additional file 1: Fig. 1. Among the patients with no history of HCC, the MELD score could be evaluated in 379 patients. Similarly, those with a MELD score of ≤ 7 points had a better prognosis than did those with higher scores Additional file 2: Fig. 2. However, the small number of cases prevented us from performing multivariate analysis.

## Discussion

Data on the clinical course of decompensated HCV-related cirrhosis in Asian patients are limited. This study shows that these patients have a favorable prognosis, possibly due to symptomatic treatment. Particularly important for a good long-term prognosis were albumin infusion for hypoalbuminemia, administration of diuretics for ascites and lower limb edema, ammonia-lowering therapy for the prevention and treatment of hepatic encephalopathy, antibacterial treatment for infectious diseases, and prophylactic treatment for esophageal varices (endoscopic variceal ligation or endoscopic injection sclerotherapy).

In our study, the median follow-up was 2 years, and the dropout rate was low (3.0%). A previous study [8] reported that liver cancer treatment improves prognosis by enhancing and maintaining adequate liver reserve. Globally, the most commonly reported cause of death is liver failure (including hepatorenal syndrome and sepsis) (17%), followed by variceal hemorrhage (10%) and HCC (5%) [13]. In Japan, liver cancer was the major cause of death [6]. And the same result was obtained in our study. The actuarial probability of HCC development in patients with decompensated HCV-related cirrhosis was higher in our study than that in a previous report (5-year rate: 41.3% vs 29.7%) [8]. The high rate of HCC may reflect the larger elderly population with preserved liver function in Japan than in overseas countries, suggesting that the risk of HCC development progressively increases. The American Association for the Study of Liver Diseases (AASLD) recommends the use of systemic therapy compared to no therapy for patients with Child–Pugh A cirrhosis or well-selected patients with Child–Pugh B cirrhosis plus advanced HCC with macrovascular invasion and/or metastatic disease. Recently, there has been increased interest in agents targeting programmed cell death protein 1 (PD-1) and its ligands (PD-L1 and PD-L2). Such agents have a significant impact on the treatment outcomes of traditionally difficult-to-treat diseases such as melanoma and non-small-cell lung cancer, among others. Immune checkpoint inhibitors targeted against cytotoxic T-lymphocyte antigen-4, PD-1, and PD-1 ligand are being investigated as a combination therapy [14]. According to the AASLD guidelines, molecular-targeted drug treatment and immune checkpoint inhibitors are recommended for Child–Pugh B, even in decompensated cirrhosis. In the future, even for HCC with decompensated cirrhosis, improvement of the prognosis of life expectancy in the decompensated phase should be the goal through provision of therapeutic intervention at an earlier stage. Curative liver cancer treatment is difficult in decompensated cirrhosis. However, the prognosis of decompensated cirrhosis can be prolonged by continuing optimal treatment while comprehensively judging the patient’s performance status (PS), hepatic reserve, and tumor factors. In this study, liver cancer was the most important cause of death for decompensated cirrhosis. However, the small number of cases examined for both liver cancer treatment and prognosis according to the MELD score prevented us from performing multivariate analysis. Furthermore, it was difficult to make an accurate evaluation for each liver cancer treatment method due to the difference in the background of the Barcelona Clinic Liver Cancer stage. These issues need to be addressed in future studies.

The MELD score is undoubtedly more objective than the Child Pugh score because it is calculated based on the etiology of cirrhosis using three simple and reproducible laboratory parameters (INR, serum creatinine, and bilirubin levels [15, 16]. In our study, PT-INR could only be evaluated in 68% of all cases; thus, we excluded them from the statistical analysis. For reference, in cases that could be evaluated, PT-INR was a significant predictor of prognosis in univariate analysis (*P* < 0.001, log-rank test), In our results, the eGFR and serum sodium level were important factors as components of the MELD score. Platelets reflect the degree of liver fibrosis. The AFP level reflects not only liver cancer, but also severe inflammation and fibrosis progression [6–8].

The Cirrhosis Acute GastroIntestinal Bleeding (CAGIB) score also includes four laboratory variables (i.e., total bilirubin [TBIL], albumin, serum creatinine [Scr], and alanine aminotransferase [ALT]). Inclusion of TBIL, albumin, and Scr into this new model is essential because they are important components of the conventional scoring systems (i.e., MELD and Child–Pugh scores). A CAGIB score greater than − 4.6646 suggests a high risk of in-hospital death in patients with liver cirrhosis and acute gastrointestinal bleeding [17]. In our study, we were only able to evaluate diabetes mellitus in 30% of all cases; therefore, we excluded them from the statistical analysis. For reference, in cases that could be evaluated, the CAGIB score was a significant predictor of prognosis in univariate analysis (*P* < 0.001, log-rank test). We hope to evaluate more recent prognostic models, including CAGIB score, in future studies.

In patients with decompensated cirrhosis, albumin reduces systemic inflammation and cardiocirculatory dysfunction [18], prolongs overall survival when administered long-term, and is a potential disease-modifying treatment [19]. Recent evidence shows that it also prevents complications, eases the management of ascites, and reduces the number of hospitalizations, thus making it cost effective [20]. In general, short-term use of albumin in decompensated cirrhosis is mainly aimed at maintaining or improving volemia. On the other hand, recent reports have the opposite report, and although there are both arguments for and against on that subject, it is the topic for future study [21].

It is important to examine the prognostic impact of antiviral therapy. To the extent that it can be considered, cases with prior interferon (IFN) treatment may have a better prognosis than do those without IFN treatment (data not shown); however, the number of cases examined was inadequate for a multivariate analysis. In addition, the combination of the DAA drugs sofosbuvir (an NS5B polymerase inhibitor) and velpatasvir (a novel NS5A inhibitor) inhibit HCV RNA synthesis [22, 23]. Velpatasvir was the first drug to be approved for decompensated HCV-related cirrhosis in Japan. Its efficacy and safety in patients with type C decompensated cirrhosis were confirmed in a domestic phase III study [22]. In that study, 26% of the patients who achieved a sustained viral response for ≥ 12 weeks showed improvement in terms of Child–Pugh classification (C to B, 33%; B to A, 25%). Hence, the prognosis of patients with Child–Pugh B and C cirrhosis can be improved with DAA treatment. In this study, the prognostic effect of antiviral therapy was not been investigated and should thus be evaluated in future research.

This study has some limitations. First, the results may differ from those obtained in prospective studies owing to the single-center retrospective cohort design. Second, we did not conduct a detailed analysis of the treatments provided during the study period. Prospective trials are needed to validate our findings.

## Conclusions

Among Japanese patients with decompensated hepatitis C virus-related cirrhosis, HCC is the most common liver-related event and is associated with poor prognosis. Knowledge of liver-related events in Japanese patients with decompensated HCV-related cirrhosis is essential. Importantly, our results highlight the importance of managing liver-related events, including hepatocellular carcinoma, in these patients. These findings can be helpful for defining the natural course and prognosis of decompensated HCV-related cirrhosis and for clinical decision-making.

## Supplementary Information

**Additional file 1**. Figure 1. Cumulative 15-year survival rates by MELD score in patients with decompensated HCV-related cirrhosis (N = 969). The rates differed significantly between patients with MELD score ≧ 7 and < 7 (log-rank test, *P* = 0.0012). HCV, hepatitis C virus. MELD, Model for End-stage Liver Disease.

**Additional file 2**. Figure 2. Cumulative 15-year survival rates by MELD score in patients with decompensated HCV-related cirrhosis without HCC (N = 379). The rates differed significantly between patients with MELD score ≧ 7 and < 7 (log-rank test,* P* = 0.0017). HCV, hepatitis C virus. MELD, Model for End-stage Liver Disease.

## Data Availability

The datasets generated and/or analyzed in the present study are available from the corresponding author on reasonable request.

## Abbreviations

AASLD: American Association for the Study of Liver Diseases
AFP: α-Fetoprotein
ALT: Alanine aminotransferase
CAGIB: Cirrhosis acute gastrointestinal bleeding
DAA: Direct-acting antiviral
eGFR: Estimated glomerular filtration rate
GGT: γ-Glutamyl transpeptidase
HCC: Hepatocellular carcinoma
HCV: Hepatitis C virus
IFN: Interferon
MELD: Model for end-stage liver disease
PD-1: Programmed cell death protein-1
PHGB: Portal hypertensive gastrointestinal bleeding
Scr: Serum creatinine
SPSS: Statistical package for the social sciences
TBIL: Total bilirubin
