# Supplementary material for: Mortality rates and risk factors in 1412 Japanese patients with decompensated hepatitis C virus-related cirrhosis: a retrospective long-term cohort study
Source: BMC Gastroenterol. 2021 Apr 23;21:189. doi: 10.1186/s12876-021-01770-0 (PMC8066946; doi:10.1186/s12876-021-01770-0)
Supplement: Supplementary file 2 — Additional file 2. Figure 2. Cumulative 15-year survival rates by MELD score in patients with decompensated HCV-related cirrhosis without HCC (N = 379). The rates differed significantly between patients with MELD score ≧ 7 and < 7 (log-rank test, P = 0.0017). HCV, hepatitis C virus. MELD, Model for End-stage Liver Disease. [file 12876_2021_1770_MOESM2_ESM.pptx]

## Slide 1
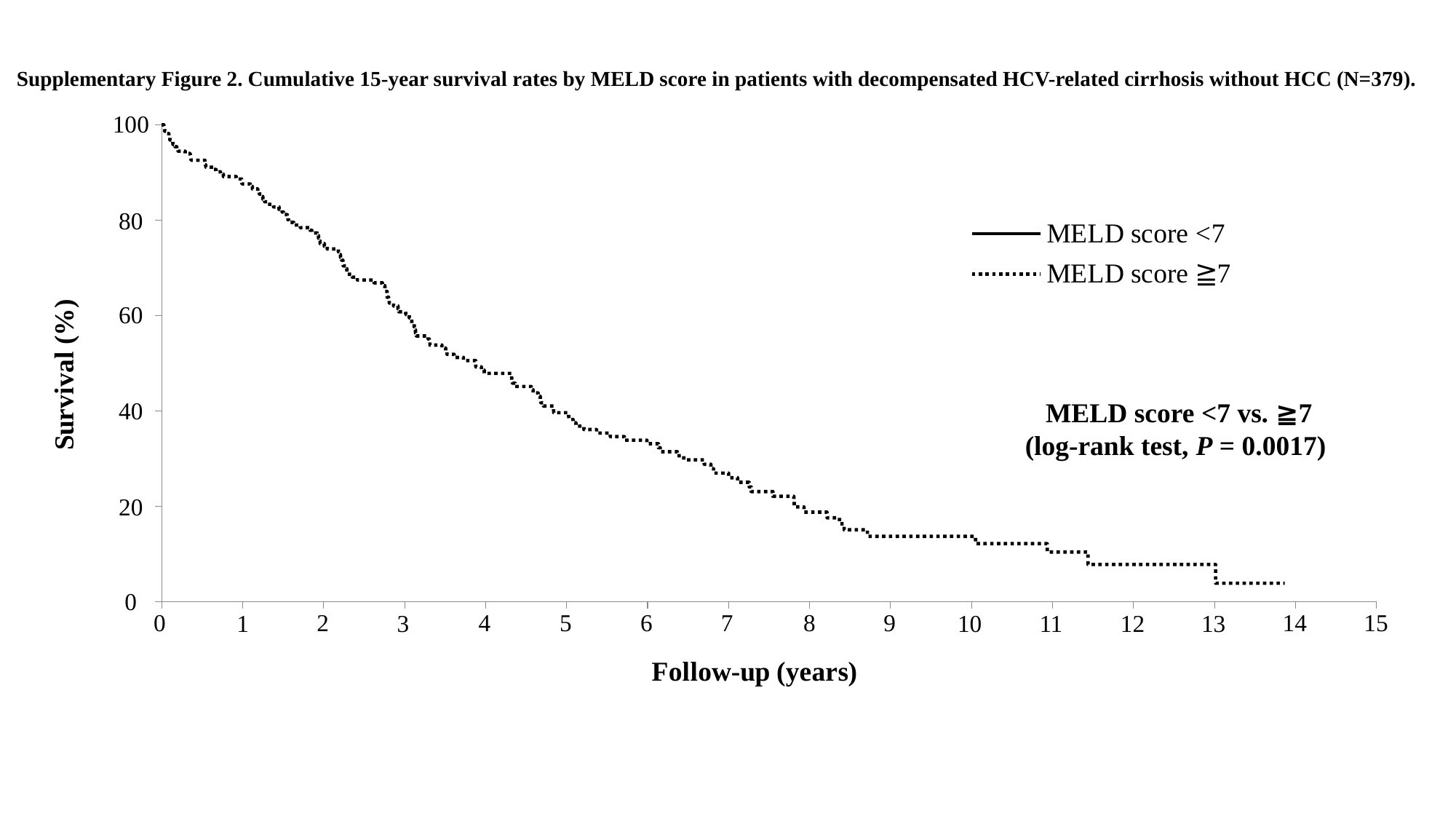

Supplementary Figure 2. Cumulative 15-year survival rates by MELD score in patients with decompensated HCV-related cirrhosis without HCC (N=379).
### Chart
| Category | MELD score <7 | MELD score ≧7 |
|---|---|---|100
80
60
40
MELD score <7 vs. ≧7
(log-rank test, P = 0.0017)
20
0
14
15
4
5
6
7
8
9
0
2
1
3
11
12
10
13
